# Correlations of P53, SLC7A11, CD3+ T cells, and CD8+ T cells with pathological features and prognosis in endometrial carcinoma: Insights from an Asian cohort

**DOI:** 10.5937/jomb0-60128

**Published:** 2026-03-17

**Authors:** Yuanjie Lv, Weiwei Zhou, Jingyu Zhang, Jing Feng, Rongge Xing, Zhigang Miao

**Affiliations:** 1 Cangzhou Central Hospital, Department of Pathology, Cangzhou, China; 2 Cangzhou Central Hospital, Department of Gynecology, Cangzhou, China

**Keywords:** p53, SLC7A11, CD3+ T cells, CD8+ T cells, endometrial carcinoma, diagnosis, p53, SLC7A11, CD3+ T ćelije, CD8+ T ćelije, karcinom endometrijuma, dijagnoza

## Abstract

**Background:**

This study investigates the clinical significance of Solute Carrier Family 7 Member 11 (SLC7A11), Tumor Protein p53 (p53), c D3+ T cells, and CD8+ T cells in endometrial carcinoma (EC). By evaluating their expression patterns and correlations with histopathological parameters and patient outcomes, we aim to develop a prognostic risk model integrating these biomarkers.

**Methods:**

We enrolled 134 EC patients (observation group) diagnosed between February 2023 and January 2024 and 128 concurrent healthy controls, and were confirmed to have no endometrial abnormalities by gynecological examination and ultrasound. Quantitative and qualitative assessments were conducted to measure SLC7A11, p53, and CD3+/CD8+ T cell levels. Diagnostic performance of individual and combined markers was analyzed using receiver operating characteristic (ROC) curves, while a logistic regression-based predictive model was developed. Associations of these biomarkers with EC histopathological features and 1-year survival outcomes were further evaluated.

**Results:**

Compared to controls, EC patients exhibited notable reductions in CD3+/CD8+ T-cell infiltration but elevations in SLC7A11 and p53 expression (P&lt;0.05). According to qualitative analysis, CD3 and CD8 positivity were similarly lower in tumor tissues than in adjacent normal tissue, whereas SLC7A11 and p53 staining were more frequent in malignant lesions (P &lt;0.05). For EC diagnosis, the combined AUC of p53, SLC7A11, CD3+ T cells, and CD8+ T cells reached 0.883 (72.4% sensitivity, 88.3% specificity). Their combination also effectively predicted 1-year mortality risk (AUC = 0.889), with 82.4% sensitivity and 81.0% specificity.

**Conclusions:**

p53, SLC7A11, CD3+ T cells, and CD8+ T cells show intimate connections with EC. A diagnostic model incorporating these biomarkers enhances the accuracy of EC detection, offering improved clinical utility.

## Abstract (sr)

**Uvod:**

Ova studija istražuje klinički značaj Solute Carrier Family 7 Member 11 (SLC7A11), Tumor Protein p53 (p53), CD3+ T ćelija i CD8+ T ćelija kod endometrijalnog karcinoma (EC). Procenom njihovih obrazaca ekspresije i korelacija sa histopatološkim parametrima i ishodima pacijenata, cilj NAM je da razvijemo prognostički model rizika koji integriše ove biomarkere.

**Metode:**

Uključili smo 134 pacijentkinje sa endometrijalnim karcinomom (grupa za posmatranje) kod kojih je dijagnostikovan između februara 2023. i januara 2024. godine i 128 istovremenih zdravih kontrolnih žena, za koje je ginekološkim pregledom i ultrazvukom potvrđeno da nemaju abnormalnosti endometrijuma. Sprovedene su kvantitativne i kvalitativne procene radi merenja nivoa SLC7A11, p53 i CD3+/CD8+ T celija. Dijagnostičke performanse pojedinačnih i kombinovanih markera analizirane su korišćenjem ROC krivih, dok je razvijen prediktivni model zasnovan na logističkoj regresiji. Dalje su procenjene povezanosti ovih biomarkera sa histopatološkim karakteristikama endometrijalnog karcinoma i ishodima preživljavanja nakon jedne godine.

**Rezultati:**

U poređenju sa kontrolama, pacijenti sa endometrijalnim karcinomom pokazali su značajno smanjenje infiltracije CD3+/CD8+ T-ćelija, ali povećanje ekspresije SLC7A11 i p53 (P&lt;0,05). Prema kvalitativnoj analizi, pozitivnost CD3 i CD8 bila je slično niža u tumorskim tkivima nego u susednom normalnom tkivu, dok su bojenje SLC7A11 i p53 bila češća u malignim lezijama (P&lt;0,05). Za dijagnozu EK, kombinovana AUC (ploća pod krivom) p53, SLC7A11, CD3+ T ćelija i CD8+ T ćelija dostigla je 0,883 (senzitivnost 72,4%, specifičnost 88,3%). Njihova kombinacija je takođe efikasno predvidela rizik od smrtnosti u roku od 1 godine (AUC = 0,889), sa osetljivošću 82,4% i specifičnošću 81,0%.

**Zaključak:**

p53, SLC7A11, CD3+ T ćelije i CD8+ T ćelije pokazuju bliske veze sa endometrijalnim karcinomom (EC). Dijagnostički model koji uključuje ove biomarkere poboljšava tačnost detekcije EC, nudeći poboljšanu kliničku korisnost.

## Introduction

As a leading gynecologic malignancy worldwide, endometrial cancer (EC) has seen a notable rise in cases in recent years, especially in perimenopausal and estrogen-exposed populations [1]. Although conventional pathological classifications (e.g., endometrioid and serous carcinoma) offer fundamental diagnostic insights, they often fail to capture tumor heterogeneity or predict prognosis accurately [2]. In the era of precision medicine, molecular markers have emerged as key tools for enhancing EC characterization and management [3]. For instance, Solute Carrier Family 7 Member 11 (SLC7A11) upregulation inhibits ferroptosis while stimulating glutathione synthesis, correlating with increased tumor mutation load and altered immunotherapy sensitivity [4]. Meanwhile, CD3^+^/CD8^+^ T-cell infiltration, a key measure of antitumor immunity, influences chemoradiotherapy efficacy [5]. Additionally, the Tumor Protein p53 (p53) exerts bidirectional control over cancer growth by maintaining genomic stability via cell cycle regulation, DNA repair, and apoptosis [6]. Current international guidelines now mandate incorporating molecular marker status into the standard diagnostic and therapeutic workflow for EC, highlighting their clinical significance. Nevertheless, existing research on SLC7A11 and p53 predominantly involves Western populations [7][8], with no validation studies examining potential expression differences in Asian populations. Moreover, research has yet to determine if multi-marker analysis improves predictive performance over single markers, or combined use with clinical-pathological parameters enables better risk stratification.

To address these limitations, this study will systematically analyze the relationship betweenSLC7A11, p53, CD3^+^ T cells, and CD8^+^ T cells andEC, and establish a unique EC risk assessment model incorporating these biomarkers. These research strategies break through the limitations of traditional single-factor analyses, providing novel perspectives on tumor-immune dynamics. Furthermore, our Asian-centric study design enhances its clinical relevance for EC management across China, Korea, Japan, and other Asia-Pacific populations.

## Materials and methods

### Research subjects

Based on the sample size estimation results of G-Power software [effect size = 0.3, = 0.05, power (1-β) = 0.8], this study enrolled 134 EC patients (observation group) admitted from February 2023 to January 2024 and 128 concurrent healthy people (control group). EC patients were eligible if they: (1) had pathological confirmation, (2) were aged 18-70, (3) provided complete clinicopathologic data, (4) had preserved tumor samples (surgically resected) for immunohistochemistry/molecular testing, and (5) had full follow-up records. The healthy controls included had no significant medical history or abnormal physical findings. Exclusion criteria covered other cancers, cardiovascular/autoimmune diseases, and preoperative anticancer treatments (radiotherapy/chemotherapy/targeted therapy). The healthy controls included had no significant medical history (including gynecological diseases) or abnormal physical findings, and were confirmed to have no endometrial abnormalities by gynecological examination and ultrasound. Exclusion criteria for healthy controls: history of any malignancy, autoimmune disorders, chronic inflammatory conditions, or use of immunosuppressive medications.The study protocol received ethical approval, and informed consent was obtained from all participants.

### Laboratory examinations

Quantitative analysis: SLC7A11 and p53 expression in peripheral blood was detected by Quantitative Real-Time Polymerase Chain Reaction (qRT-PCR). 2-5 mL of whole blood was diluted with an equal volume of PBS and centrifuged with Ficoll- Paque density gradient (2000 rpmx20 min) to collect peripheral blood mononuclear cells (PBMCs). Following total RNA isolation using a TRIzol reagent kit (Invitrogen, USA), reverse transcription into cDNA was carried out as per kit instructions (Takara Bio, Japan). PCR reactions were then conducted in a system comprising 2xSYBR Green Master Mix (10 μL) + upstream primer (0.4 mL) + downstream primer (0.4 μL) + cDNA template + DEPC water (7.2 μL) (Cycling parameters: 95 /5 s, 60 /30 s, 72 /30 s, 40 cycles) (Takara Bio, Japan). 2^"ΔΔCI^ calculated SLC7A11 and p53 expression against GAPDH (see Table 1 for primer sequences, designed and constructed by Jiangsu Saisuofei Biotechnology Co., LTD., China). Peripheral blood CD3^+^ T cells, and Cd8^+^ T cells were then quantitatively analyzed by flow cytometry. 100 μL of whole blood was lysed with a red blood cell lysis buffer (BD FACS Lysing Solution), incubated at room temperature for 10 min, and centrifuged (300xgx5 min), followed by supernatant removal and resuspension in PBS. Next, each tube received 100 μL of cell suspension + antibody combination [CD3 (FITC-Clone UCHT1), CD8 (PE-Clone RPA-T8)] (BioLegend, USA). After a 20-minute room-temperature incubation in darkness, samples were processed by flow cytometry. FSC/SSC gating was applied for cellular debris and non-viable cell elimination, followed by automated data acquisition. Instrument calibration was performed prior to testing using BD Cytometer Setup & Tracking beads to verify consistent laser power, flow speed, and compensation settings. Routine fluidic system maintenance was conducted to minimize contamination risks.

**Table 1:** Primer sequences.

	F (5'-3')	R (5'-3')	bp
SLC7A11	GCTGCTGGTGATGGTGTT G	TGGTCAGCAGGTCAGGTAG	150 bp
p53	AGGCGCAAGAAGATGATG	TCAGTCTGAGTCGGACCTT	142 bp
GAPDH	GAAG GTGAAG GTCGGAGTC	GAAGATGGTGATGGGATTTC	226 bp

Qualitative analysis (Immunohistochemistry-IHC): Fresh surgically resected EC tissue and normal tissue adjacent to the cancer were obtained from EC patients. IHC was performed to assess the protein expression and localization of SLC7A11 and p53 within the tumor microenvironment. The samples were fixed in 10% neutral formalin solution for 48h, paraffin-embedded, and sliced (4 μm). Immunohistochemical staining was performed utilizing SLC7A11 (EP1555Y) and p53 (DO-7 clone) antibodies (Abcam, USA) after deparaffinization and rehydration. Staining was visualized using DAB, followed by hematoxylin counterstaining. Positive expression, indicated by the presence of yellow-brown stained cells, was identified microscopically. A semi-quantitative scoring system (H-score) was used: staining intensity (0: none, 1: weak, 2: moderate, 3: strong) multiplied by the percentage of positive cells (0-100%). Samples with an H-score ≥ 5 were considered positive for SLC7A11 and p53.

### Follow-up for prognosis

EC patients were monitored for at least one year, with mandatory monthly follow-ups (median: 13 [9-16] months). The endpoint was defined as patient death, with January 2025 set as the cutoff. One-year prognostic survival was recorded.

### Statistical methods

Data analysis was performed with SPSS 24.0. The comparison of qualitative data [n(%)] uses chi- square test. Quantitative data distribution was assessed using the Shapiro-Wilk test: normally distributed data (χ̄±s) underwent independent t-tests, whereas non-normal data [M (P25, P75)] were evaluated with the Mann-Whitney U test. Receiver Operating Characteristic (ROC) curves identified diagnostic thresholds (including sensitivity and specificity) by maximizing the Youden index, with Area Under the Curve (AUC) quantifying accuracy. A combined detection formula was generated from logistic regression coefficients. A *P*-value below 0.05 was considered statistically significant.

## Results

### Baseline data comparison between control and observation groups

A comparative assessment of patients' baseline data like age and family history was conducted, which revealed no statistical significance and thus confirmed their comparability (*P*>0.05). Meanwhile, the standardized mean difference (SMD) analysis revealed negligible differences in baseline characteristics between groups (all SMD values <0.1), demonstrating effective covariate balance and minimizing the influence of potential confounding factors (Table 2).

**Table 2:** Clinical baseline data.

	Control group	Observation group	t(or 2)	P	SMD
Age	57.66±7.39	58.70±5.45	1.308	0.192	0.0065
BMI (kg/m^2^)	25.27±2.73	25.00±2.16	0.883	0.378	0.0030
Family history of EC			1.007	0.316	0.0054
Yes	18 (14.1)	25 (18.7)			
No	110 (85.9)	109 (81.3)			
Underlying chronic<br>diseases					
Diabetes	58 (45.3)	56 (41.8)	0.330	0.566	0.0057
Hypertension	48 (37.5)	54 (40.3)	0.216	0.642	0.0046
Hyperlipidemia	26 (20.3)	30 (22.4)	0.168	0.682	0.0041
Smoking			0.950	0.330	0.0009
Yes	26 (20.3)	30 (22.4)			
No	102 (79.7)	100 (74.6)			
Drinking			0.477	0.490	0.0069
Yes	18 (14.1)	23 (17.2)			
No	110 (85.9)	111 (82.8)			
Reproductive history			0.628	0.428	0.0079
Yes	116 (90.6)	125 (93.3)			
No	12 (9.4)	9 (6.7)			

### Diagnostic value of SLC7A11, p53, CD3^+^ T cells, and CD8^+^ T cells quantitation in EC

**Figure 1:**
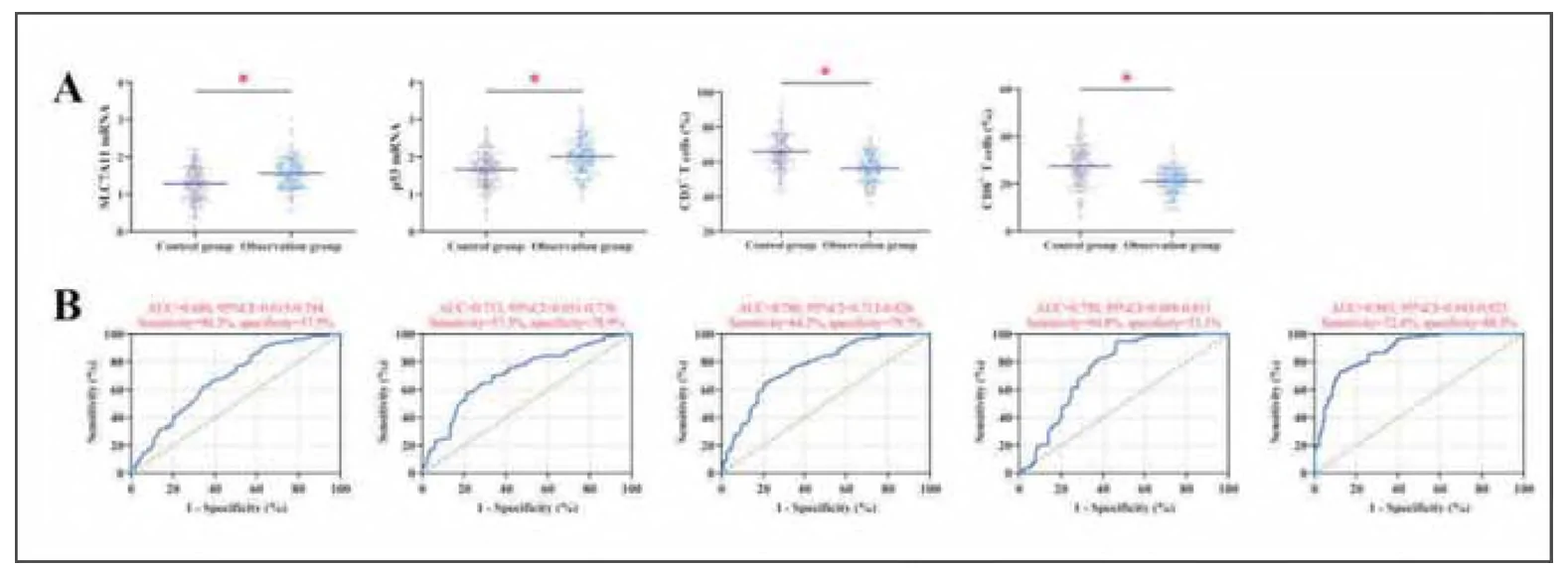
Diagnostic value of SLC7A11, p53, CD3^+^ T cells, and CD8^+^ T cells quantitation in EC. A: Comparison of SLC7A11, p53 mRNAs, CD3^+^ T cells, and CD8^+^ T cells between the two groups of subjects. B: ROC curves of SLC7A11, p53 mRNAs, CD3^+^ T cells, and CD8^+^ T cells for the diagnosis of EC. * indicates P < 0.05.

Compared with controls, EC cases exhibited reduced CD3^+^ T cells, and CD8^+^ T cells as well as elevated SLC7A11 and p53 expression (*P*>0.05, Figure 1A). ROC curve analysis indicated the diagnostic value of SLC7A11, p53, and CD3^+^/CD8^+^ T cell quantification for EC, though with an AUC range of merely 0.680-0.780 (Figure 1B). Subsequent regression analysis established significant correlations between these markers and EC pathogenesis (Table 3). The combined detection formula developed in this study achieved superior performance (72.4% sensitivity, 88.3% specificity, AUC = 0.883), demonstrating significant improvement over single-marker approaches (Figure 1B).

**Table 3:** Association of SLC7A11, p53 mRNAs, CD3^+^ T cells, and CD8^+^ T cells and EC (Logistic regression analysis). Note: »-« means not applicable; regression coefficient, B; standard error, S.E.; odds ratio, OR; confidence interval, CI.

	SLC7A11	p53	CD3^+^ T cells	CD8^+^ T cells	Constant
B	1.783	1.532	-0.111	-0.114	4.920
S.E.	0.440	0.389	0.020	0.029	1.626
Wals χ^2^	16.418	15.541	31.763	25.321	9.160
OR	5.947	4.627	0.895	0.866	-
95%CI	2.510-14.087	2.160-9.910	0.861-0.930	0.819-0.916	-
* P *	<0.001	<0.001	<0.001	<0.001	-

### Relationship between SLC7A11 and p53 qualitative analysis and EC pathological features

The results of qualitative analysis revealed increased positive rates of SLC7A11 and p53 positivity in EC tissues (*P*<0.05). Further analysis of pathological characteristics revealed no pathological types- related differences in SLC7A11 and p53 positivity rates (*P*>0.05). The positive rate of p53 in patients with tumor diameter >3 cm was higher than that in patients with tumor diameter 3 cm (*P*=0.035). The positive rates of SLC7A11 and p53 were increased in patients with poor differentiation, lymph node metastasis, and myometrial invasion ≥1/2 (*P*<0.05, Table 4).

**Table 4:** Relationship between SLC7A11, p53 qualitative analysis and EC pathological features. Note: * since the frequency is less than 5, Fisher's precision probability test is used, »-« means not applicable.

	n	SLC7A11	p53
Sample			
Peritumoral tissue	134	42 (31.3)	48 (35.8)
Tumor tissue	134	92 (68.7)	110 (82.1)
* χ^2^*		37.308	59.273
* P *		<0.001	<0.001
Pathological type			
Endometrioid adenocarcinoma	94	64 (68.1)	76 (80.9)
Mucinous/serous adenocarcinoma	40	28 (70.0)	34 (85.0)
* χ^2^*		0.048	0.329
* P *		0.827	0.567
Tumor diameter (cm)			
≤3	86	56 (65.1)	66 (76.7)
>3	48	36 (75.0)	44 (91.7)
* χ^2^*		1.398	-
* P *		0.237	0.035*
Differentiation			
Poorly	41	34 (82.9)	38 (92.7)
Moderately/well	93	58 (62.4)	72 (77.4)
* χ^2^*		5.590	-
* P *		0.018	0.049*
Lymph node metastasis			
Yes	38	32 (84.2)	36 (94.7)
No	96	62 (62.5)	74 (77.1)
* χ^2^*		5.963	-
P		0.015	0.022*
Myometrial invasion			
<1/2	88	54 (61.4)	67 (76.1)
≥1/2	46	38 (82.6)	43 (93.5)
* χ^2^*		6.336	-
* P *		0.012	0.016*

### Diagnostic value of SLC7A11, p53, CD3^+^ T cells, and CD8^+^ T cells in EC prognosis

**Figure 2:**
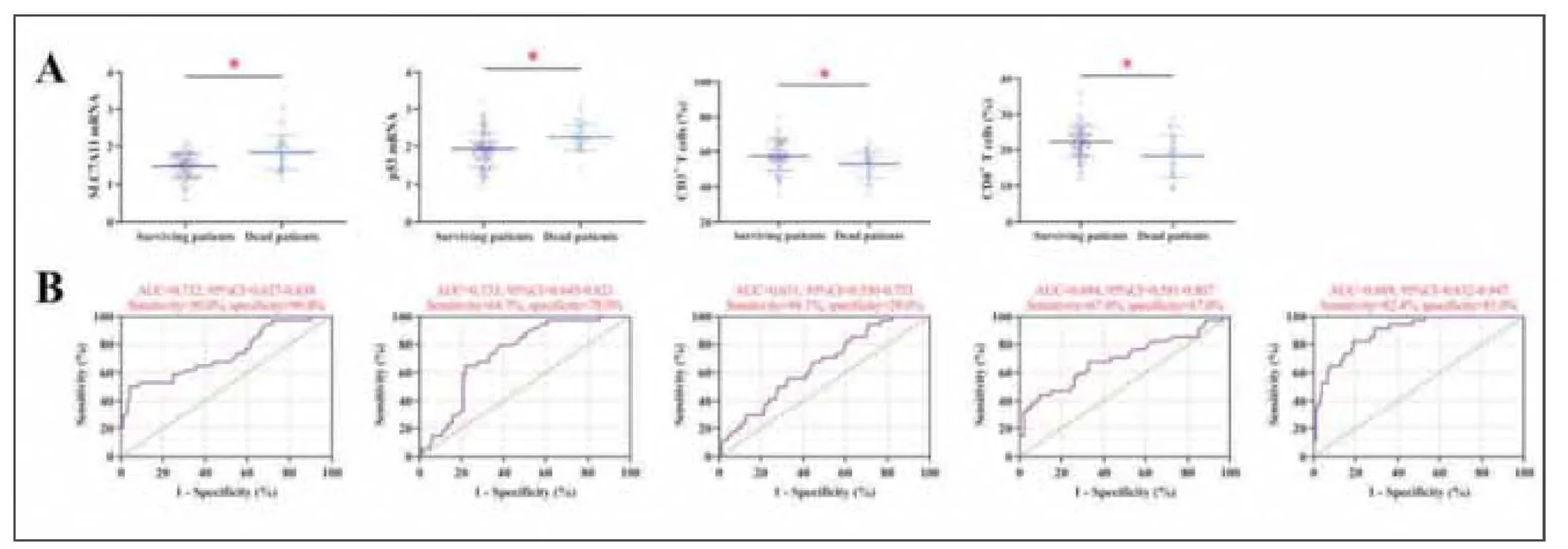
Diagnostic value of SLC7A11, p53, CD3^+^ T cells, and CD8^+^ T cells in EC prognosis. A: Comparison of SLC7A11, p53 mRNAs, CD3^+^ T cells, and CD8^+^ T cells between the surviving and dead patients. B: ROC curves of SLC7A11, p53 mRNAs, CD3^+^ T cells, and CD8^+^ T cells for the diagnosis of prognostic death. * indicates P < 0.05.

All EC patients completed follow-up, with 34 deaths (25.37% mortality). Comparative analysis demonstrated significant reductions in CD3^+^ T cells, and CD8^+^ T cells in deceased patients and concurrent upregulation of p53 and SLC7A11 (*P*<0.05, Figure 2A). In addition, individual biomarkers showed limited 1-year mortality prediction (AUC range 0.651-0.733), whereas their combination achieved superior prognostic performance (AUC 0.889, 82.4% sensitivity, 81.0% specificity; *P*<0.05, Table 5, Figure 2B).

**Table 5:** Association of SLC7A11, p53 mRNAs, CD3^+^ T cells, and CD8^+^ T cells and EC prognosis (Logistic regression analysis). Note: »-« means not applicable.

	SLC7A11	p53	CD3^+^ T cells	CD8^+^ T cells	Constant
B	3.461	2.253	-0.079	-0.176	-3.450
S.E.	0.900	0.721	0.034	0.058	2.785
Wals 2	14.784	9.758	5.600	9.234	1.535
OR	31.836	9.514	0.924	0.839	-
95%CI	5.455-18.789	2.315-39.102	0.865-0.986	0.749-0.940	-
P	<0.001	0.002	0.018	0.002	-

## Discussion

EC management is evolving with molecular typing and immune microenvironment insights. While traditional pathological classification systems serve as morphological diagnostic tools, their capacity to predict tumor heterogeneity and treatment response remains rather restricted [9]. Recent advancements, including TCGA-based molecular classification, have significantly enhanced prognostic evaluation by incorporating SLC7A11 expression and p53 mutation data [10]. This pioneering study in Chinese EC patients examines SLC7A11, p53, and CD3^+^/CD8^+^ T cell expression, linking them to clinical outcomes to guide localized precision treatment.

In this study, we found upregulated SLC7A11 in EC patients than in healthy controls, consistent with previous research findings [11]. Existing studies have confirmed the ability of SLC7A11 to promote EC cell invasiveness and metastasis through STAT3/NF- B pathway activation and EMT-related transcription factor (e.g., Snail and Slug) upregulation [12], which emphasizes the close relationship between SLC7A11 and the pathological progression of EC. Besides, by maintaining redox homeostasis, high SLC7A11 expression may weaken the killing effect of chemotherapy drugs on tumor cells and reduce chemosensitivity [13], suggesting SLC7A11's utility in assessing treatment response. However, qualitative analysis reveals a lower prevalence of SLC7A11-positive EC cases in Asian patients than in Western reports [14], hinting at potential ethnic differences in molecular profiles. Additionally, p53 expression abnormalities in EC were found to correlate significantly with tumor grade and lymph node metastasis. Despite its recognized tumor-suppressive role, p53 mutations cause abnormal protein accumulation, driving malignancy [15]. Mutant p53 drives tumor progression through loss of tumor-suppressive activity, with its overexpression strongly linked to worse outcomes in serous and high-grade (G3) endometrioid carcinomas [16][17]. Of particular note, a notable difference in p53 mutation rates was observed between poorly (92.7%) and well-differentiated EC (76.7%), implying that p53 mutations may be the key driving factor of EC progression. Furthermore, p53 and SLC7A11 are known to be important co-transcribed genes. In EC, p53 inactivation has been reported to potentially upregulate SLC7A11 via metabolic reprogramming [18], suggesting a possible 'mutant-p53-SLC7A11' axis that may accelerate malignancy synergistically. However, as our study did not include mechanistic experiments, this interaction remains to be fully elucidated in the context of endometrial cancer. Of course, these insights also support the rationale for combined detection. The decrease in CD3^+^ T cells, and CD8^+^ T cells in EC patients is probably related to their role in anti-tumor immune response. As we all know, CD3^+^ T cells are the core subgroup of tumor-infiltrating lymphocytes, reflecting adaptive immune response intensity. CD8+ T cells function as cytotoxic effectors that directly kill tumors, with their infiltration level closely related to patient prognosis [19][20]. Gallego A et al. similarly confirmed the positive connection between CD3^+^ T cells, and CD8^+^ T cells and survival in EC patients [21], which can support our results. The elevated p53 levels in our study further suggest that tumor immune escape may involve p53-p21 pathway-mediated T cell inhibition [22].

Then, through ROC curve analysis, we found that while individual markers (SLC7A11: 0.680, p53: 0.713, CD3^+^: 0.780, CD8^+^: 0.750) showed moderate diagnostic value, their combination dramatically improved performance (AUC=0.883, 72.4% sensitivity, 88.3% specificity). This marked improvement over single-marker approaches highlights the complementarity of multi-dimensional molecular models. Moreover, our analyses revealed that the composite model's predictive accuracy (AUC 0.889) for one-year survival outcomes significantly outperformed all individual parameters (AUCs between 0.651 to 0.733). These findings undoubtedly provide new reference and guidance for EC assessment. It is worth noting that in this study, we use quantitative analysis, as it not only facilitates sample collection but also generates continuous variable data versus qualitative approaches, thus more accurately reflecting tumor microenvironment biology. Meanwhile, this enables the construction of clinically actionable risk stratification systems, where diagnostic thresholds guide both patient categorization and subsequent treatment personalization.

Of course, the limitations of this study can not be ignored. One is the small sample size (n=134 EC patients) and the single-center retrospective design, resulting in possible selection bias. Another drawback is the insufficient long-term survival data due to the restricted follow-up time. Moreover, the lack of in vitro experiments leads to the inability to determine the exact mechanism of SLC7A11, p53, CD3^+^ T cells, and CD8^+^ T cells in EC. These issues need to be confirmed by further research with further expanded samples, extended follow-up periods, and the integration of multi-omics data to build a more accurate prediction model.

## Conclusion

CD3^+^ T cells, and CD8^+^ T cells are decreased in EC patients, while p53 and SLC7A11 are increased. SLC7A11, p53, CD3^+^ T cells, and CD8^+^ T cells are closely related to the pathological characteristics of EC. The combined detection model integrating the four biomarkers significantly improves the diagnostic and prognostic evaluation accuracy for EC through quantitative analysis, providing a novel approach for identifying optimal candidates for immunotherapy and optimizing risk stratification.

## Dodatak

### Ethical approval

The study involving human subjects complied with the Declaration of Helsinki and was approved by the ethical committee of Cangzhou Central Hospital, and all participants provided written informed consent.

### Availability of data and materials

The data used to support the findings of this study are available from the corresponding author upon request.

### Funding

Not applicable.

### Conflict of interest statement

All the authors declare that they have no conflict of interest in this work.
